# Frequency of Hypoxemia and Hyperoxemia and Their Association With 90-Day Mortality in Critically Ill Oncology Patients: A Retrospective Observational Study

**DOI:** 10.7759/cureus.111644

**Published:** 2026-06-28

**Authors:** Asad Aleem, Yusra Saleem, Sadia Sadaqat, Amina Abid

**Affiliations:** 1 Anesthesiology, Shaukat Khanum Memorial Cancer Hospital and Research Centre, Lahore, PAK; 2 Anesthesia and Critical Care, Shaukat Khanum Memorial Cancer Hospital and Research Centre, Lahore, PAK; 3 Internal Medicine, Shaukat Khanum Memorial Cancer Hospital and Research Centre, Lahore, PAK

**Keywords:** hyperoxemia, hypoxemia, intensive care, mortality, oxygen therapy

## Abstract

Background: Oxygen therapy is a cornerstone of critical care management. Both inadequate oxygenation and excessive oxygen exposure have been associated with adverse physiologic effects and poorer clinical outcomes. While hypoxemia may lead to tissue hypoxia and organ dysfunction, hyperoxemia has been implicated in oxidative stress and cellular injury. This study aimed to determine the frequency of hypoxemia and hyperoxemia among critically ill patients and evaluate their association with 90-day mortality.

Methods: A retrospective observational study was conducted, including 228 critically ill oncology patients admitted to the intensive care unit (ICU) or high-dependency unit (HDU). The frequency of hypoxemia and hyperoxemia was determined using predefined criteria. Ninety-day mortality was the primary outcome. Associations between oxygenation abnormalities and mortality were evaluated using chi-square and Fisher's exact tests. Univariable and multivariable logistic regression analyses were performed to identify independent predictors of mortality after adjustment for age, sex, and ICU/HDU admission status and oxygenation variables.

Results: A total of 228 critically ill patients were included in the analysis, with 90-day mortality observed in 126 patients (55.3%). There were no significant differences in age or sex between survivors and non-survivors. Patients who died were more frequently admitted to the ICU than the HDU and had a significantly higher prevalence of hypoxemia. Hyperoxemia was numerically more common among non-survivors. Mortality was markedly higher among patients with hypoxemia compared with those without, and was also higher among hyperoxemic patients. On univariable analysis, ICU admission, hypoxemia, and hyperoxemia were all associated with increased odds of 90-day mortality. After adjustment for age, sex, ICU/HDU status, and oxygenation status, increasing age, ICU admission, and hypoxemia remained independently associated with mortality, whereas hyperoxemia was not. Male sex was not associated with mortality. However, association with severe illness scores like Acute Physiology and Chronic Health Evaluation (APACHE) and Sequential Organ Failure Assessment (SOFA) scores was not analyzed and remains a major limitation of the study.

Conclusion: Hypoxemia was common among critically ill patients and associated with increased 90-day mortality. Mortality increased progressively with worsening hypoxemia severity. Hyperoxemia demonstrated an association with mortality on unadjusted analysis but was not independently associated after adjustment. However, these findings should be interpreted with caution because illness severity scores were unavailable and could not be incorporated into the analysis.

## Introduction

Patients admitted to critical care settings worldwide represent a heterogeneous population with diverse primary diagnoses and varying combinations of comorbid conditions, reflecting the complexity of critical illness and its management. The most common causes for admission to the intensive care unit (ICU) and high-dependency unit (HDU) include hemodynamic instability, respiratory failure, and neurological complications [1]. Oxygen administration is an essential component of critical care medicine and is frequently used to maintain adequate tissue oxygenation in critically ill patients. Previous research has demonstrated that hypoxemia and hyperoxemia occur in the critically ill irrespective of their diagnosis and treatment [2,3]. Both these conditions can have deleterious effects on the patient’s prognosis. Evidence supports targeted oxygen therapy based on oxygen saturation and arterial oxygen tension to minimize the risks associated with both hypoxemia and hyperoxemia and improve clinical outcomes; however, oxygenation targets should be individualized according to the patient's clinical condition, underlying disease, and risk factors rather than relying on a single universal target for all critically ill patients [3]. Although oxygen therapy is lifesaving, both insufficient and excessive oxygen exposure may have harmful consequences.

Hypoxemia and hyperoxemia are defined on the basis of arterial oxygen tension and PaO₂/FiO₂ ratio as mentioned in the methods section. Hypoxemia may impair cellular metabolism and result in organ dysfunction through inadequate oxygen delivery [4]. Persistent hypoxemia has been associated with increased morbidity and mortality in critically ill populations; on the other hand, hyperoxemia has increasingly attracted attention as a potentially harmful state. Excess oxygen exposure may promote reactive oxygen species formation, vasoconstriction, oxidative stress, and inflammatory injury, thereby contributing to adverse clinical outcomes [5].

Previous studies investigating the relationship between oxygen abnormalities and mortality have produced mixed findings [6]. While several investigations have demonstrated increased mortality associated with hypoxemia and hyperoxemia, the independent contribution of these abnormalities remains uncertain and may vary according to illness severity and patient population [5-7].

Critically ill oncology patients represent a unique population because malignancy-related physiological derangements, treatment-related toxicities, immunosuppression, and frequent respiratory complications may influence oxygen requirements and outcomes. Despite widespread oxygen use in this setting, data regarding the prevalence of hypoxemia and hyperoxemia and their association with mortality among critically ill oncology patients remain limited [8]. Therefore, evaluation of oxygenation abnormalities in this population may provide clinically relevant information regarding oxygen management and its effects.

The present study aimed to determine the frequency of hypoxemia and hyperoxemia among critically ill oncology patients and evaluate their association with 90-day mortality. We additionally examined whether increasing hypoxemia severity was independently associated with mortality.

## Materials and methods

Study design and setting

This retrospective observational study was conducted using data from patients admitted to the ICU and HDU at Shaukat Khanum Memorial Cancer Hospital and Research Centre (SKMCH&RC), Lahore, Pakistan. Institutional Review Board approval and waiver of informed consent were obtained prior to data collection and analysis (IRB letter no. EX-25-03-25-01).

Study population

ICU and HDU admissions were screened for eligibility during the final six months of 2024. After application of predefined exclusion criteria, 228 patients were included in the final analysis. Eligible patients included both adult and pediatric patients, intubated and non-intubated, admitted to ICU or HDU settings for at least 24 hours. As the ICU and HDU at our institution provide care for both adult and pediatric oncology patients, both populations were included to reflect the full spectrum of critically ill oncology patients managed at our center. Patients having incomplete arterial blood gas data, missing mortality follow-up, ICU stay less than 24 hours, and repeat admissions were excluded.

Data collection and definitions

Demographic and clinical variables collected included age, sex, admission location, oxygenation status, hypoxemia severity, and 90-day mortality extracted from electronic medical records. Hypoxemia was defined as a PaO₂/FiO₂ ratio of ≤300 mmHg and classified as mild (201-300 mmHg), moderate (101-200 mmHg), and severe (≤100 mmHg) [2]. Hyperoxemia was defined as PaO₂ >100 mmHg, consistent with previously published critical care literature evaluating excess oxygen exposure [3]. "PaO₂" refers to the arterial partial pressure of oxygen measured in mmHg, and "FiO₂" refers to the fraction of inspired oxygen administered to the patient. Oxygenation status was determined using the first arterial blood gas obtained within 24 hours of ICU/HDU admission. This approach was selected to ensure standardized classification of oxygen status across all patients. Ninety-day mortality was predefined as the primary study outcome and was recorded after evidence of death within ninety days from ICU admission as per the hospital records.

Statistical analysis

Continuous variables were assessed for normality and are presented as mean ± standard deviation (SD), while categorical variables are presented as frequencies and percentages. Comparisons between survivors and non-survivors were performed using the independent-samples t-test for continuous variables and the chi-square or Fisher's exact test for categorical variables, as appropriate. Univariable logistic regression was initially performed to evaluate crude associations between clinical variables and 90-day mortality. Variables considered clinically relevant or associated with mortality in univariable analyses were subsequently entered into a multivariable logistic regression model to identify independent predictors of mortality. Adjusted odds ratios with 95% confidence intervals were reported. The final multivariable model included age, sex, admission location, hypoxemia, and hyperoxemia. Relative risks were additionally calculated to provide clinically interpretable estimates of mortality associated with oxygenation abnormalities. Statistical significance was defined as a two-sided p-value <0.05.

## Results

**Figure 1:**
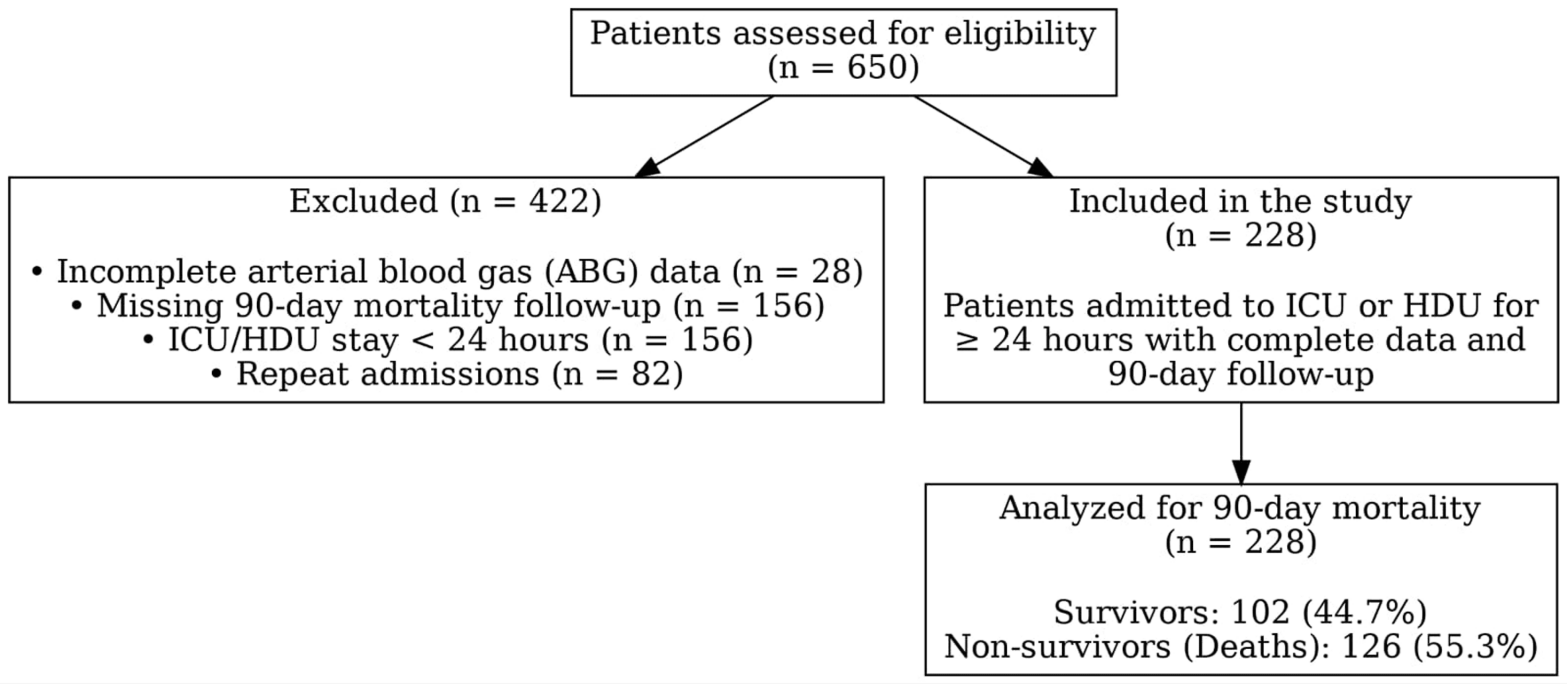
STROBE flow diagram showing patient screening, exclusions, inclusion, and 90-day mortality assessment ICU: intensive care unit; HDU: high-dependency unit; STROBE: Strengthening the Reporting of Observational Studies in Epidemiology

During the study period, 650 ICU and HDU admissions were screened for eligibility. After application of predefined exclusion criteria, 228 patients were included in the final analysis (Figure 1).

Ninety-day mortality occurred in 126 patients (55.3%), while 102 patients (44.7%) survived. Hypoxemia was observed in 118 patients (51.8%), and hyperoxemia occurred in 45 patients (19.7%).

There was no significant difference in age between survivors and non-survivors (30.5 ± 20.9 vs 34.4 ± 20.3 years; t = -1.41, P = 0.159). Similarly, sex distribution was comparable between the two groups (χ² = 0.29, P = 0.589). Patients who died were significantly more likely to have been admitted to the ICU rather than the HDU (94.4% vs 68.6%; χ² = 24.71, P < 0.001). Hypoxemia was substantially more common among non-survivors compared with survivors (71.4% vs 27.5%; χ² = 41.92, P < 0.001). Hyperoxemia was more frequently observed among patients who died (24.6% vs 13.7%); however, this difference did not reach statistical significance (χ² = 3.55, P = 0.059) (Table 1).

**Table 1:** Baseline characteristics according to 90-day mortality ICU: intensive care unit; SD: standard deviation; χ²: chi-square value; t: t-test value

Variable	Survivors (n=102)	Non-survivors (n=126)	Test statistic	P-value
Age (years), mean ± SD	30.5 ± 20.9	34.4 ± 20.3	t = -1.41	0.159
Male sex, n (%)	57 (55.9)	76 (60.3)	χ² = 0.29	0.589
ICU admission, n (%)	70 (68.6)	119 (94.4)	χ² = 24.71	<0.001
Hypoxemia, n (%)	28 (27.5)	90 (71.4)	χ² = 41.92	<0.001
Hyperoxemia, n (%)	14 (13.7)	31 (24.6)	χ² = 3.55	0.059

Mortality among patients with hypoxemia was 76.3%, compared with 32.7% among patients without hypoxemia. Mortality among hyperoxemic patients was 68.9%, compared with 51.9% among patients without hyperoxemia (Table 2).

**Table 2:** Mortality according to oxygenation status

Variable	Deaths n/N (%)	Survivors n/N (%)
Hypoxemia	90/118 (76.3)	28/118 (23.7)
No hypoxemia	36/110 (32.7)	74/110 (67.3)
Hyperoxemia	31/45 (68.9)	14/45 (31.1)
No hyperoxemia	95/183 (51.9)	88/183 (48.1)

On univariable logistic regression analysis, ICU admission (OR (odds ratio) 7.84, 95% CI 3.29-18.69, P < 0.001), hypoxemia (OR 6.68, 95% CI 3.74-11.95, P < 0.001), and hyperoxemia (OR 2.12, 95% CI 1.06-4.23, P = 0.034) were associated with increased odds of 90-day mortality (Table 3).

**Table 3:** Univariable logistic regression analysis for 90-day mortality ICU: intensive care unit; OR: odds ratio; CI: confidence interval

Variable	Crude OR	95% CI	P-value
Age (per year increase)	1.009	0.996–1.021	0.188
Male sex	1.216	0.717–2.063	0.469
ICU admission	7.84	3.29–18.69	<0.001
Hypoxemia	6.68	3.74–11.95	<0.001
Hyperoxemia	2.12	1.06–4.23	0.034

After adjustment for age, sex, ICU/HDU status, hypoxemia, and hyperoxemia, increasing age remained independently associated with mortality (adjusted OR 1.018, 95% CI 1.003-1.034, P = 0.021). ICU admission remained strongly associated with mortality (adjusted OR 7.709, 95% CI 2.872-20.692, P < 0.001). Hypoxemia remained independently associated with increased odds of mortality (adjusted OR 6.388, 95% CI 3.414-11.954, P < 0.001). Hyperoxemia was not independently associated with mortality after adjustment (adjusted OR 1.946, 95% CI 0.886-4.274, P = 0.097). Male sex was not associated with mortality (Table 4).

**Table 4:** Multivariable logistic regression analysis for 90-day mortality ICU: intensive care unit; CI: confidence interval

Variable	Adjusted OR	95% CI	P-value
Age (per year increase)	1.018	1.003–1.034	0.021
Male sex	1.104	0.588–2.071	0.759
ICU admission	7.709	2.872–20.692	<0.001
Hypoxemia	6.388	3.414–11.954	<0.001
Hyperoxemia	1.946	0.886–4.274	0.097

Mortality increased progressively with worsening hypoxemia severity. Mortality was 60.5% in patients with mild hypoxemia, 81.5% in patients with moderate hypoxemia, and 88.5% in patients with severe hypoxemia (Table 5).

**Table 5:** Mortality according to hypoxemia severity

Hypoxemia Severity	Deaths/Total n/N	Mortality (%)
Mild	23/38	60.5
Moderate	44/54	81.5
Severe	23/26	88.5

Relative risks (RRs) with 95% confidence intervals were calculated for mortality associated with hypoxemia and hyperoxemia (Table 6).

**Table 6:** Relative risk of mortality according to oxygenation status CI: Confidence interval

Exposure	Relative Risk	95% CI
Hypoxemia	2.33	1.75-3.10
Hyperoxemia	1.33	1.04-1.69

## Discussion

This study demonstrated three principal findings. First, hypoxemia was highly prevalent among critically ill patients admitted to ICU and HDU settings. Second, hypoxemia was associated with increased 90-day mortality even after adjustment for demographic variables and admission location. Third, although hyperoxemia demonstrated an association with mortality on unadjusted analysis, this association was no longer statistically significant after multivariable adjustment.

The observed association between hypoxemia and mortality is consistent with previous literature demonstrating the harmful effects of inadequate oxygen delivery in critically ill populations [8-10]. Persistent hypoxemia may contribute to tissue hypoxia, impaired cellular metabolism, multiorgan dysfunction, and death. Mortality increased progressively with worsening hypoxemia severity, suggesting a graded association between oxygenation impairment and adverse outcomes.

It is worth mentioning that although age did not differ significantly between survivors and non-survivors on unadjusted analysis, it remained independently associated with mortality after adjustment, suggesting confounding in crude comparisons.

Hyperoxemia has increasingly been recognized as a potentially harmful physiologic state due to oxidative stress, vasoconstriction, and inflammatory injury [8,9].In this study, hyperoxemia was associated with numerically higher crude mortality. Although the simple unadjusted group comparison did not reach statistical significance, hyperoxemia was associated with increased odds of mortality in univariable logistic regression; however, this association was attenuated after multivariable adjustment, which differs from recent published data, especially in a cohort of critically ill oncology patients [8]. This may be due to the presence of confounding factors, such as the unavailability of illness severity scores and the assessment of their association with the primary outcome. This suggests that the apparent relationship between hyperoxemia and mortality may be influenced by illness severity or ICU admission status rather than representing an independent determinant of outcome. Hyperoxemia may therefore act as a marker of greater illness severity, higher oxygen requirements, or more intensive monitoring rather than a direct determinant of mortality. This interpretation should be considered when evaluating the observed association.

These findings are broadly consistent with previous investigations and systematic reviews reporting increased mortality among hypoxemic critically ill patients and mixed evidence regarding hyperoxemia. While several studies have demonstrated increased mortality associated with excessive oxygen exposure, others have reported weaker or non-significant associations after adjustment for illness severity and clinical confounding [5-7,10-13].

Published data have shown that targeted oxygen therapy is additionally beneficial in terms of decreasing morbidity and mortality, decreasing hospital and ICU stay, and improving overall physiological outcomes for critically ill patients [9-15].

Strengths of this study include its evaluation of both hypoxemia and hyperoxemia within a real-world cohort of critically ill patients admitted to ICU and HDU settings. The use of 90-day mortality as a clinically meaningful outcome strengthens the relevance of these findings. Multivariable logistic regression was performed to adjust for available confounding factors, allowing assessment of associations with mortality while recognizing that residual confounding may remain. Additionally, stratification of hypoxemia severity provided further insight into the graded relationship between oxygenation impairment and adverse outcomes. This study contributes valuable regional data from a tertiary oncology center in Pakistan, where published critical care evidence remains relatively limited. To our knowledge, this is among the few studies evaluating both hypoxemia and hyperoxemia simultaneously in a cohort of critically ill oncology patients from South Asia and reporting associations with 90-day mortality.

This study has several limitations as well. Its retrospective observational design limits causal inference and may be susceptible to residual confounding. Illness severity scores such as Acute Physiology and Chronic Health Evaluation (APACHE) [16] and Sequential Organ Failure Assessment (SOFA) [17] were unavailable and therefore could not be incorporated into adjusted analyses; the exclusion of this data is a major confounding factor, and so is the inclusion of both adult and pediatric patients, which may have introduced heterogeneity and may limit comparability with studies evaluating exclusively adult ICU populations. Oxygenation status was determined using the first arterial blood gas obtained within 24 hours of ICU/HDU admission; hence, cumulative oxygen exposure, duration of hypoxemia, duration of hyperoxemia, and temporal fluctuations in oxygenation could not be assessed, which becomes particularly relevant for hyperoxemia, as adverse effects are believed to be related to both the magnitude and duration of oxygen exposure. Additionally, the absence of time-to-event data precluded survival analyses such as Kaplan-Meier or Cox regression modeling. Finally, this was a single-center study performed on a subset of critically ill oncology patients, which may limit generalizability to broader mixed medical and surgical ICU populations.

## Conclusions

Hypoxemia was independently associated with increased 90-day mortality after adjustment for available demographic and clinical variables, although residual confounding by illness severity cannot be excluded. Increasing hypoxemia severity conferred progressively greater mortality risk. Although hyperoxemia showed an association with mortality on unadjusted analysis, it was not independently associated following adjustment, which is in contrast with recently published data. These findings highlight the importance of vigilant oxygen monitoring and individualized oxygen therapy, although causal relationships cannot be inferred from this observational study.
